# Supplementary material for: Causal Relationship between Aging and Anorexia Nervosa: A White-Matter-Microstructure-Mediated Mendelian Randomization Analysis
Source: Biomedicines. 2024 Aug 16;12(8):1874. doi: 10.3390/biomedicines12081874 (PMC11351342; doi:10.3390/biomedicines12081874)
Supplement: Supplementary file 1 [file biomedicines-12-01874-s001.zip › Supplementary Figure/Figure S4/Singe SNP plot of MR analysis for TL to ubm-b-2116.pdf]

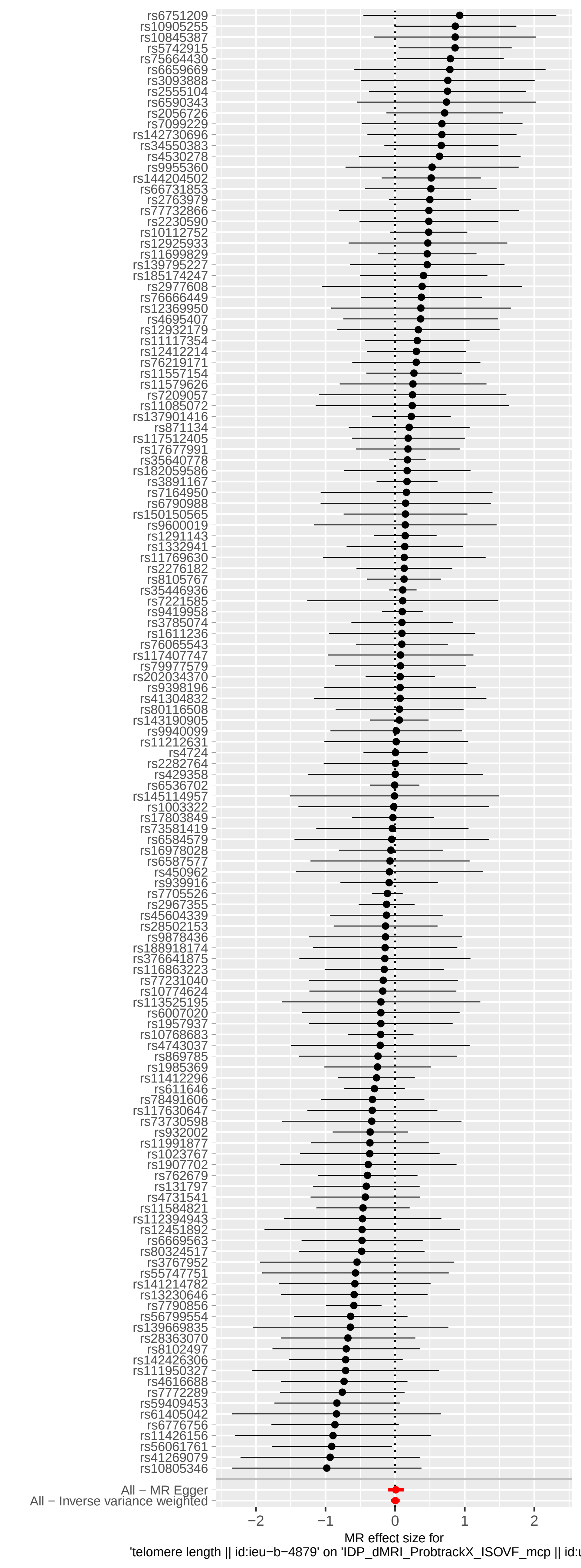
rs6751209
rs10905255
rs10845387
rs5742915
rs75664430
rs6659669
rs3093888
rs2555104
rs6590343
rs2056726
rs7099229
rs142730696
rs34550383
rs4530278
rs9955360
rs144204502
rs66731853
rs2763979
rs77732866
rs2230590
rs10112752
rs12925933
rs11699829
rs139795227
rs185174247
rs2977608
rs76666449
rs12369950
rs4695407
rs12932179
rs11117354
rs12412214
rs76219171
rs11557154
rs11579626
rs7209057
rs11085072
rs137901416
rs871134
rs117512405
rs17677991
rs35640778
rs182059586
rs3891167
rs7164950
rs6790988
rs150150565
rs9600019
rs1291143
rs1332941
rs11769630
rs2276182
rs8105767
rs35446936
rs7221585
rs9419958
rs3785074
rs1611236
rs76065543
rs117407747
rs79977579
rs202034370
rs9398196
rs41304832
rs80116508
rs143190905
rs9940099
rs11212631
rs4724
rs2282764
rs429358
rs6536702
rs145114957
rs1003322
rs17803849
rs73581419
rs6584579
rs16978028
rs6587577
rs450962
rs939916
rs7705526
rs2967355
rs45604339
rs28502153
rs9878436
rs188918174
rs376641875
rs116863223
rs77231040
rs10774624
rs113525195
rs6007020
rs1957937
rs10768683
rs4743037
rs869785
rs1985369
rs11412296
rs611646
rs78491606
rs117630647
rs73730598
rs932002
rs11991877
rs1023767
rs1907702
rs762679
rs131797
rs4731541
rs11584821
rs112394943
rs12451892
rs6669563
rs80324517
rs3767952
rs55747751
rs141214782
rs13230646
rs7790856
rs56799554
rs139669835
rs28363070
rs8102497
rs142426306
rs111950327
rs4616688
rs7772289
rs59409453
rs61405042
rs6776756
rs11426156
rs56061761
rs41269079
rs10805346
All − MR Egger
All − Inverse variance weighted
−2
−1
0
1
2
MR effect size for
'telomere length || id:ieu−b−4879' on 'IDP_dMRI_ProbtrackX_ISOVF_mcp || id:u
